# Identification of Secretory Leukoprotease Inhibitor As an Endogenous Negative Regulator in Allergic Effector Cells

**DOI:** 10.3389/fimmu.2017.01538

**Published:** 2017-11-13

**Authors:** Shintaro Matsuba, Toshiki Yabe-Wada, Kazuya Takeda, Tetsuya Sato, Mikita Suyama, Toshiyuki Takai, Toshiaki Kikuchi, Toshihiro Nukiwa, Akira Nakamura

**Affiliations:** ^1^Department of Immunology, Kanazawa Medical University, Kahoku Uchinada, Ishikawa, Japan; ^2^Division of Immunology, Faculty of Medicine, Tohoku Medical and Pharmaceutical University, Sendai, Japan; ^3^Division of Bioinformatics, Medical Institute of Bioregulation, Kyushu University, Fukuoka, Japan; ^4^Department of Experimental Immunology, Institute of Development, Aging and Cancer, Tohoku University, Sendai, Japan; ^5^Department of Respiratory Medicine and Infectious Diseases, Niigata University Graduate School of Medical and Dental Sciences, Niigata, Japan; ^6^Department of Respiratory Medicine, Tohoku University Graduate School of Medicine, Sendai, Japan

**Keywords:** secretory leukoprotease inhibitor, basophil, eosinophil, Elk-1, JNK-interacting protein 3 scaffold protein

## Abstract

Mast cells, basophils, and eosinophils are central effectors in allergic inflammatory disorders. These cells secrete abundant serine proteases as well as chemical mediators and cytokines; however, the expression profiles and functions of their endogenous inhibitors remain elusive. We found that murine secretory leukoprotease inhibitor (SLPI) is expressed in basophils and eosinophils but in not in mast cells. SLPI-deficient (*Slpi*^−/−^) basophils produce more cytokines than wild-type mice after IgE stimulation. Although the deletion of SLPI in basophils did not affect the release of chemical mediators upon IgE stimulation, the enzymatic activity of the serine protease tryptase was increased in *Slpi*^−/−^ basophils. Mice transferred with *Slpi*^−/−^ basophils were highly sensitive to IgE-mediated chronic allergic inflammation. Eosinophils lacking SLPI showed greater interleukin-6 secretion and invasive activity upon lipopolysaccharide stimulation, and the expression of matrix metalloproteinase-9 by these eosinophils was increased without stimulation. The absence of SLPI increases JNK1 phosphorylation at the steady state, and augments the serine phosphorylation of JNK1-downstream ETS transcriptional factor Elk-1 in eosinophils upon stimulation. Of note, SLPI interacts with a scaffold protein, JNK-interacting protein 3 (JIP3), that constitutively binds to the cytoplasmic domain of toll-like receptor (TLR) 4, suggesting that SLPI controls Elk-1 activation *via* binding to JIP3 in eosinophils. Mice transferred with *Slpi*^−/−^ eosinophils showed the exacerbation of chitin-induced allergic inflammation. These findings showed that SLPI is a negative regulator in allergic effector cells and suggested a novel inhibitory role of SLPI in the TLR4 signaling pathways.

## Introduction

Mast cells, basophils, and eosinophils are central effector cells in allergic diseases, but have different characteristics and functions (1–5). Mast cells are tissue resident cells that play a pivotal role in immediate hypersensitivity. The aggregation of high-affinity receptor for IgE (FcεRI) bound to IgE leads to mast cell activation, such as cytokine production and chemical mediator release (1–3). In contrast, basophils and eosinophils are present in the peripheral circulation and rapidly migrate to the sites of inflammation upon antigen stimulation. Basophils have a similar phenotype to mast cells, including the expression of FcεRI and the secretion of type 2 T helper (Th2) cytokines; however, they constitute a distinct lineage and have unique features (1–4). In particular, basophils abundantly produce interleukin (IL)-4 in response to allergens, which directly promotes Th2 differentiation (4, 5) and leads to eosinophilic inflammation *via* the activation of innate lymphoid cells (6).

Eosinophils are also major effectors in the pathogenesis of allergic disorders (7–9). Eosinophils secrete diverse inflammatory cytokines and lipid mediators, such as leukotriene (LT) C_4_ (7–9), and produce matrix metalloproteinases (MMPs), which cleaves the constituents of the basement membrane, leading to inflammatory cellular trafficking (7). Unlike other effectors, eosinophils display cytolytic activities, including extracellular trap cell death (9, 10).

In addition to cytokines and chemical mediators, these allergic effector cells synthesize and store serine proteases (7, 11). Most typical proteases are serine proteases, which are classified into chymases and tryptases (7, 11). While mast cells strongly express various types of chymases, several tryptases, such as mast cell protease (MCP) 8 and 11, are present at higher levels in basophils (11). Tryptases induce inflammatory responses *via* the degradation of fibrinogen, and chymases disrupt the tight junction, leading to the transmigration of leukocytes through the endothelium (11); however, the expression and function of serine protease inhibitors in allergic effector cells remains unknown.

Secretory leukoprotease inhibitor (SLPI) is a 12 kDa small protein produced by secretory cells and immune cells (12–17). In a steady state, SLPI is located in the granules and cytoplasm, whereas it moves into the nucleus as well as the cell surface upon stimulation (15). SLPI inhibits serine proteases, including tryptase and chymase, and protects tissues from excessive protease activity at the sites of inflammation *in vivo* (13–17). Previous studies have indicated that SLPI is a multifunctional protein that possesses anti-inflammatory as well as anti-microbial activities (14–17). In particular, SLPI regulates IκBα/β degranulation (18–20), and suppresses inflammatory cytokine responses *via* the repression of nuclear factor-κB (NF-κB) activation by binding to NF-κB consensus sites after lipopolysaccharide (LPS) stimulation (21). Although recent studies suggested the involvement of SLPI in bronchial asthma (22, 23), the expression and the exact role of SLPI in allergic effectors, mast cells, basophils, and eosinophils, remains unclear.

In the present study, we found—for the first time—that SLPI is expressed in murine basophils and eosinophils, but not in mast cells. Bone marrow (BM)-derived basophils and eosinophils from *Slpi*^−/−^ mice were highly responsive to IgE or LPS stimulation, generating increased cytokine production. *Slpi*^−/−^ eosinophils showed an increased MMP-9 gene transcription, with or without stimulation. *Slpi*^−/−^ eosinophils displayed enhanced JNK1 activation in the steady state; their serine phosphorylation of Elk-1 was also found to be enhanced in response to LPS. Surprisingly, SLPI was associated with Toll-like receptor (TLR) 4-binding scaffold JNK-interacting protein (JIP) 3, which is critical for the activation of JNK1 (24, 25). This suggests that SLPI inhibits the Elk-1 pathway by binding to JIP3. These results showed that SLPI is a novel endogenous negative regulator in allergic effector cells.

## Materials and Methods

### Mice

C57BL/6 (B6) mice were purchased from Japan SLC Inc. *Slpi*^−/−^ and *Fcer1g*^−/−^ mice have been described previously (20, 26). Congenic B6-Ly5.1 (CD45.1) mice were purchased from Sankyo Labo Service. These mice were kept and bred in the animal unit at Kanazawa Medical University and Tohoku Medical and Pharmaceutical University, an environmentally controlled and specific pathogen-free facility, in accordance with the guidelines for experimental animals defined by the facilities. All of the studies were approved by the Animal Studies Committee at Kanazawa Medical University and Tohoku Medical and Pharmaceutical University.

### Quantitative RT-PCR

Total RNA was extracted using ReliaPrep RNA Cell (Promega) or miReasy Mini kit (Qiagen), and cDNA was prepared using a ReverTra Ace qPCR RT kit (Toyobo) for reverse transcription. The gene transcript levels of mouse SLPI and MMP-9 and housekeeping S16 ribosomal protein (*RPS16*) were quantified by a real-time PCR using Go-Taq qPCR Master Mix (Promega) on a DNA Engine Opticon2 system (MJ Research). The relative amount of gene transcript was calculated and normalized by dividing the calculated value for the gene of interest by the housekeeping gene value. The PCR conditions for all genes were as follows: 95°C initial activation for 2 min, followed by 40 cycles of 95°C for 15 s, and 60°C for 60 s, and fluorescence determination at the melting temperature of the product. The primers were as follows: mouse *RPS16* forward, 5′-GATATTCGG GTCCGTG TGA-3′, and reverse, 5′-TTGAGATG GACTGTCGGATG-3′, yielding a 69-bp product; mouse *Slpi* forward, 5′-AGCCACAATGCCGTACTGACT-3′, and revere, 5′-AGGCTTCCTCCAC ACTGGTT T-3′, yielding a 115-bp product; mouse *Mmp9* forward, 5′-TGACTACGATAAGGACG GCAAA-3′, and reverse, 5′-GATGAACGGGAAC ACACAGG-3′, yielding a 100-bp product.

### The Induction of Mast Cells, Basophils, and Eosinophils from BM Cells

Basophils, eosinophils, and mast cells were derived from BM cells. The preparation of BM-derived basophils (BMBs) was carried out as described elsewhere (27). Briefly, BM cells were cultured with 5 ng/ml IL-3 (Pepro Tech) for 12 days. On Day 12, c-kit^−^ DX5^+^ cells were isolated as basophils using the MACS system with magnetic microbead-conjugated anti-DX5 antibody (Miltenyi Biotec). BM-derived eosinophils (BMEos) were induced from BM cells as previously described (28). Briefly, BM cells were cultured in the presence of 100 ng/ml stem cell factor (SCF) (Miltenyi Biotec) and 100 ng/ml FMS-like tyrosine kinase 3 ligand (Flt3L; Miltenyi Biotec) for 4 days. On Day 4, SCF and Flt3L were replaced with IL-5 (10 ng/ml; Peprotech). The cultured cells were collected and used as BMEos (purity, ≥95%) on Day 14. Mast cells were grown from BM cells as described (29). Mast cells were prepared by culturing BM cells in the presence of 5 ng/ml IL-3 for 8–12 weeks. Cellular proliferation was determined with propidium iodide staining using an ADAM-MC automatic cell counter (NanoEnTek Inc.).

### Fluorescence Microscopy and Transmission Electron Microscopy (TEM)

Cells were fixed in 4% paraformaldehyde in PBS and then treated with permeabilizing buffer (BD Bioscience). The cells were blocked with blocking reagent (Toyobo) for 1 h at room temperature, incubated with biotinylated goat anti-mouse SLPI (R&D Systems) in Can get signal solution A (Toyobo) overnight at 4°C, and then incubated with allophycocyanin-labeled streptavidin in Can get signal solution B (Toyobo) for another 1 h at room temperature. The specimens were mounted with SlowFade Gold and the nuclei were stained with 4′,6-diamino-2-phenylindole (DAPI; Thermo Fisher Scientific). Images were obtained with a BZ-9000 fluorescence microscope (Keyence). For the TEM analysis, the samples were postfixed in 1% OsO_4_ in 0.1 M sodium cacodylate buffer and embedded in Quetol 651 (Polysciences). Ultrathin sections (80 nm) of cells were cut on an Ultracut UCT ultramicrotome (Leica). Images were obtained with a TEM H-7650 (Hitachi High-Technologies).

### Flow Cytometry and Cell Sorting

Flow cytometry was conducted using the following antibodies (All purchased from Biolegend unless stated otherwise): anti-IgE (RME-1), anti-TLR4 (SA15-21), anti-Siglec-F (E50-2440; BD Bioscience), anti-CCR3 (J073E5), anti-CD11b (M1/70; BD Bioscience), anti-ST2 (DIH9), anti-FcεRIα (MAR-1), anti-CD23 (B3B4), anti-CD123 (5B11), anti-CD49b (DX5), anti-CD117 (2B8), anti-CD45.1 (A20), and anti-CD45.2 (104). Fc-mediated nonspecific staining was blocked with anti-CD16/32 (2.4G2 hybridoma culture supernatant). Events were acquired using a FACSCanto II (BD Bioscience), and the data of 10,000–100,000 events were analyzed using the FACSDiva (BD Bioscience) or FlowJo software programs (FlowJo). The surface molecule expression was calculated by defining the delta mean fluorescence intensity between the specific antibody stain and the isotype-matched control antibody. Splenic basophils and eosinophils, and peritoneal eosinophils were isolated by using a cell sorting system (SH800; Sony Biotechnology). Separation of these cells were shown in Figure S1 in Supplementary Material. Briefly, CD4^+^CD8^+^B220^+^ cells were depleted from B6 splenocytes using magnetic separator (Miltenyi Biotec). Splenic basophils were sorted by gating on FcεRIα^+^ cells from CD4^–^CD8^–^B220^−^ spleen cells. Splenic eosinophils were also sorted by gating on Siglec-F^+^ cells from CD4^–^CD8^–^B220^−^ spleen cells. Peritoneal eosinophils were isolated by gating on Siglec-F^+^ cells. The purity of the sorted populations was consistently ≥90%, as determined by the FcεRIα^+^ DX5^+^ and Siglec-F^+^ phenotypes.

### The Enzymatic Activities of Tryptase and Chymase

Bone marrow-derived basophils were incubated for 1 h at 37°C with 5 µg/ml anti-2,4,6-trinitrophenol (TNP)-IgE in 200 µl of culture medium and then stimulated for 12 h at 37°C with 1 µg/ml TNP-OVA in 200 µl of HEPES-tyrode’s buffer, pH 7.4. Eosinophils were incubated with LPS (0.1 µg/ml) for 12 h. The culture supernatants were collected, and the tryptase and chymase activities were detected by adding MeOSuc-AAPV-pNA and N-Suc-AAPF-pNA (chromogenic substrates; Sigma-Aldrich) at a final concentration of 1 mM, respectively. The absorbance was measured at 405 nm at 37°C.

### β-Hexosaminidase (HEX) Activity

β-Hexosaminidase activity was assayed as previously described (30). Briefly, 50 µl of the sample was incubated with 50 µl of 1 mM p-nitrophenyl-N-acetyl-β-d-glucosamide (Sigma-Aldrich) dissolved in 0.1 M citrate buffer (pH 5.0) in a 96-well microtiter plate at 37°C for 1.5 h. The reaction was stopped with 200 µl/well of 0.1 M NaOH/0.2 M glycine, pH 10.7, and measured at 405 nm in a plate reader. For the analysis of the total cell content of β-HEX, cells were lysed with 1.0% (vol/vol) Triton X-100 in HEPES-tyrode’s buffer. The percentage of degranulation was calculated as follows: the absorbance of culture supernatants at 405 nm/absorbance of the total cell lysate supernatants at 405 nm.

### Eosinophil Peroxidase (EPO) Activity

Bone marrow-derived eosinophils were incubated with LPS (Sigma-Aldrich), IL-33, and Phorbol 12-myristate 13-acetate (PMA)-ionomycin (Biolegend) for 12 h at the indicated concentration. The EPO activity was measured by the spectrophotometric method (31). Briefly, 100 µl of culture supernatant from each sample was placed in a 96-well plate, and 100 µl of substrate solution containing 0.1 µM o-phenylenediamine-dihydrochloride, 0.1% Triton X-100, and 1 µM hydrogen peroxide (Sigma-Aldrich) was added in each well. After incubation for 30 min at 37°C, the enzymatic reaction was stopped by adding 50 µl of 4 M sulfuric acid. Absorbance was measured at 492 nm using a Multiscan JX system (Thermo Fisher Scientific).

### ELISA

Bone marrow-derived basophils and BMEos were incubated with the indicated stimulators. The levels of cytokines and histamine in the culture supernatants were determined using ELISA kits, in accordance with the manufacturers’ instructions. The mouse IL-4, IL-6 ELISA MAX Standard kit (Biolegend), the IL-13 Ready-Set-Go ELISA set (Thermo Fisher Scientific), a Histamine ELISA test kit (Neogen), and a Cysteinyl leukotrine ELISA kit (Cayman Chemical) were used.

### Chemotaxis and Invasion Assays

Chemotaxis of BMEos was performed in 24-well chemotaxis chambers containing polycarbonate filters (pore size: 5 µm, Kurabo, Osaka, Japan). The wells of the lower chamber were filled with 5% bovine serum albumin (Wako)-RPMI1640 medium (Sigma-Aldrich) with LPS (*Escherichia coli* O111:B4; Sigma-Aldrich) and CCL11 (R&D Systems) and incubated for 2 h at 37°C. Eosinophils were then applied to the wells of the upper chambers and incubated for a further 2.5 h. The number of cells migrating from the upper chamber to the lower chamber was counted *via* the trypan blue-exclusion test. An invasion assay was performed with a BioCoat invasion system (BD Bioscience) in accordance with the manufacturer’s instructions. The insert membrane was coated with a basement membrane extract. Eosinophils (5 × 10^5^ cells) were applied to the wells of the upper chambers. The lower chambers of the 24-well plate were filled with 750 µl of serum-free RPMI1640 medium with 1 µg/ml LPS and 10 nM CCL11 and then incubated for 22 h at 37°C. The number of cells migrating from the upper chamber to the lower chamber was again counted using the trypan blue-exclusion test. The percent of invasion was calculated as the number of cells invading through the Matrigel insert membrane divided by the number of cells migrating through the control insert membrane.

### Immunoblotting and Immunoprecipitation

Cells were solubilized in lysis buffer (1.0% NP-40, 50 mM HEPES, pH7.4, 150 mM NaCl) containing protease and phosphatase inhibitor (Thermo fisher scientific). Cell lysates were separated by SDS-PAGE, transferred to a Polycinylidene difluoride (PVDF) membrane, and detected with the following antibodies using ECL substrate (Bio-Rad): rabbit anti-PLCγ2, antiphospho-PLCγ2, anti-Erk1/2, antiphospho-Erk1/2, antiphospho-JNK1, anti-Elk-1, antiphospho-Elk-1(Ser383), anti-NF-κBp65, antiphospho-NF-κBp65, mouse anti-IκBα (Cell Signaling Technology), rabbit antiphospho Elk-1 (Thr417) (Thermo Fisher Scientific), rabbit anti-NF-κBp65 mouse anti-IκBβ (Santa Cruz Biotechnology), goat antimouse SLPI, rabbit antimouse MMP-9 (R&D Systems), rabbit anti-JNK1, rat anti-mouse MCP8, rat antimouse MCP11 (Biolegend), horseradish peroxidase (HRP)-conjugated goat (Santa Cruz Biotechnology), mouse (Cell Signaling Technology), or rabbit IgG antibodies (Cell Signaling Technology). For the immunoprecipitaion analysis, cell lysates (5 × 10^6^ cells) were precleaned by Dynabeads protein G (VERITAS), and were sequentially incubated with mouse anti-JIP3 (F-6; Santa Cruz Biotechnology) or mouse IgG1κ isotype control (MG1-45; Biolegend) and Dynabeads protein G. The immunoprecipitates were detected with the following antibodies using SuperSignal™ West Pico Chemiluminescent Substrate (Thermo Fisher Scientific): mouse-anti JIP3 (F-6) or biotinylated goat-anti mouse SLPI (R&D Systems), and HRP-conjugated goat IgG antibodies (Santa Cruz Biotechnology) or streptavidin (Biolegend). Digital images were obtained using an ImageQuant LAS4000 mini instrument (GE Healthcare). Densitometry was performed on scanned blots using the ImageQuant TL software program (GE Healthcare).

### IgE-Mediated Chronic Allergic Inflammation

The protocol by which IgE-mediated chronic allergic inflammation (IgE-CAI) was previously described (27). DX5^+^ cells containing basophils were fractionated with ant-DX5 magnetic beads from B6 and *Slpi*^−/−^ mice. DX5^+^ BM cells were transferred to *Fcer1g*^−/−^ mice 4 days after 5-fluorouracil (5-FU) (Sigma-Aldrich) marrow suppression. Two days after the adaptive cell transfer, the recipient mice were intravenously injected with 2 µg of DNP-IgE (SPE-7; Sigma-Aldrich). Antigen challenge was performed in the right ear by applying 0.6% DNFB acetone-olive oil solution (20 µl). Simultaneously, an equal amount of acetone-olive oil solution was administered to the left ear. The ear thickness was measured using a dial thickness gage (Mitsutoyo). For the histological analysis, both ears were removed from euthanized mice on Day 6 after the antigen challenge and stained with HE.

### The Induction of Passive Systemic Anaphylaxis

Mouse IgE anti-TNP mAbs (C38-2) (BD Bioscience) were administered intravenously through the tail vein (volume, 100 µl/mouse). Mice were injected intravenously with 1.0 mg of TNP-OVA in saline 24 h after the injection of IgE (150 µg/mouse). Changes in the core body temperature associated with systemic anaphylaxis were monitored by measuring changes in the rectal temperature using a rectal probe coupled to a digital thermometer (Natsume Seisakusyo).

### Plasmid Construction and Transfection

A plasmid containing full-length mouse SLPI cDNA was purchased from DNAFORM. The open reading frame corresponding to SLPI with a fused 6× His-Tag at the C-terminal was amplified from a plasmid and cloned into pcDNA3.1 (Thermo Fisher Scientific). The primers were as follows: mouse SLPI forward, 5′-CCCCCGAATTCGAGAGCTCC-3′, and reverse, 5′-CACCGAGCATCTA GACTAGTGGTGATGGTGATGGTGATGATGACGACCTTCGATCATCGGGGGCA-3′. Plasmid transfection was performed using ScreenFect A (Wako), in accordance with the manufacturer’s instructions. Briefly, cells were seeded at 2 × 10^6^ cells/ml and transfected of *Slpi* plasmid DNA (0.3 µg) with ScreenFect A. The mRNA of *Slpi* and *Mmp9* were analyzed 1 day after transfection.

### Gene Expression Profiling

B6 and *Slpi*^−/−^ BMEOs were stimulated with/without LPS (1 µg/ml) for 3 h. Total RNA was purified with an miRNeasy Mini kit (Qiagen) in accordance with the manufacturer’s instructions. The purified RNA was converted to sense-strand cDNA using an Ambion WT Expression Kit (Thermo Fisher Scientific) and then labeled using an Affymetrix GeneChip WT Terminal Labeling and Controls Kit. Labeled cDNAs were hybridized onto the Affymetrix GeneChip Mouse Gene 1.0 ST Array using an Affymetrix GeneChip Hybridization, Wash, and Stain Kit, according to the manufacture’s protocols. The signals were quantified using an Affymetrix CeneChip Scanner 3000, and raw data were obtained using the Affymetrix GeneChip Command Console Software program (version 1.2.1.20). The data were normalized using the Robust Multichip Average algorithm in the “affy” package (32) of the Bioconductor project software program,^1^ after which transcript signals were calculated by log2-transformation of the normalized data. Further analyses were performed using the R software program.^2^ The data were archived in the NCBI Gene Expression Omnibus (accession number, GSE87638).^3^

### House-Dust Mite (HDM)-Induced Airway Inflammation

The model of HDM (Greer Laboratories)-induced asthma was developed as reported previously, with slight modification (33). Briefly, mice were anesthetized with isoflurane and intranasally sensitized with 1 µg of HDM on Day 0. Seven days later, they were challenged with 1 µg of HDM for 5 consecutive days. The mice were sacrificed, and their organs were dissected for a histological analysis on Day 14. BALF cells were obtained by an intratracheal injection of EDTA-containing PBS.

### Chitin-Induced Eosinophilic Inflammation by Eosinophil Adaptive Transfer

Eosinophilic lung inflammation was established with an intranasal challenge of chitin. Chitin (Sigma Aldrich) was prepared as previously described (34). Congenic B6-CD45.1 mice were intravenously transferred with eosinophils from B6 or *Slpi*^−/−^ mice (CD45.2) in accordance with the methods of a previous study (28). One hour after the eosinophil transfer, chitin (10^5^ beads) was intranasally administered to the recipient mice. BALF cells were obtained 24 h after the antigen challenge. The donor cells were distinguished from the recipient cells by anti-CD45.1/CD45.2 antibody staining.

### Statistical Analyses

The statistical significance of differences was determined using a paired Student’s *t*-test. *P* values of <0.05 were considered to indicate statistical significance.

## Results

### SLPI Is Expressed in Basophils and Eosinophils, But Not Mast Cells

**Figure 1:**
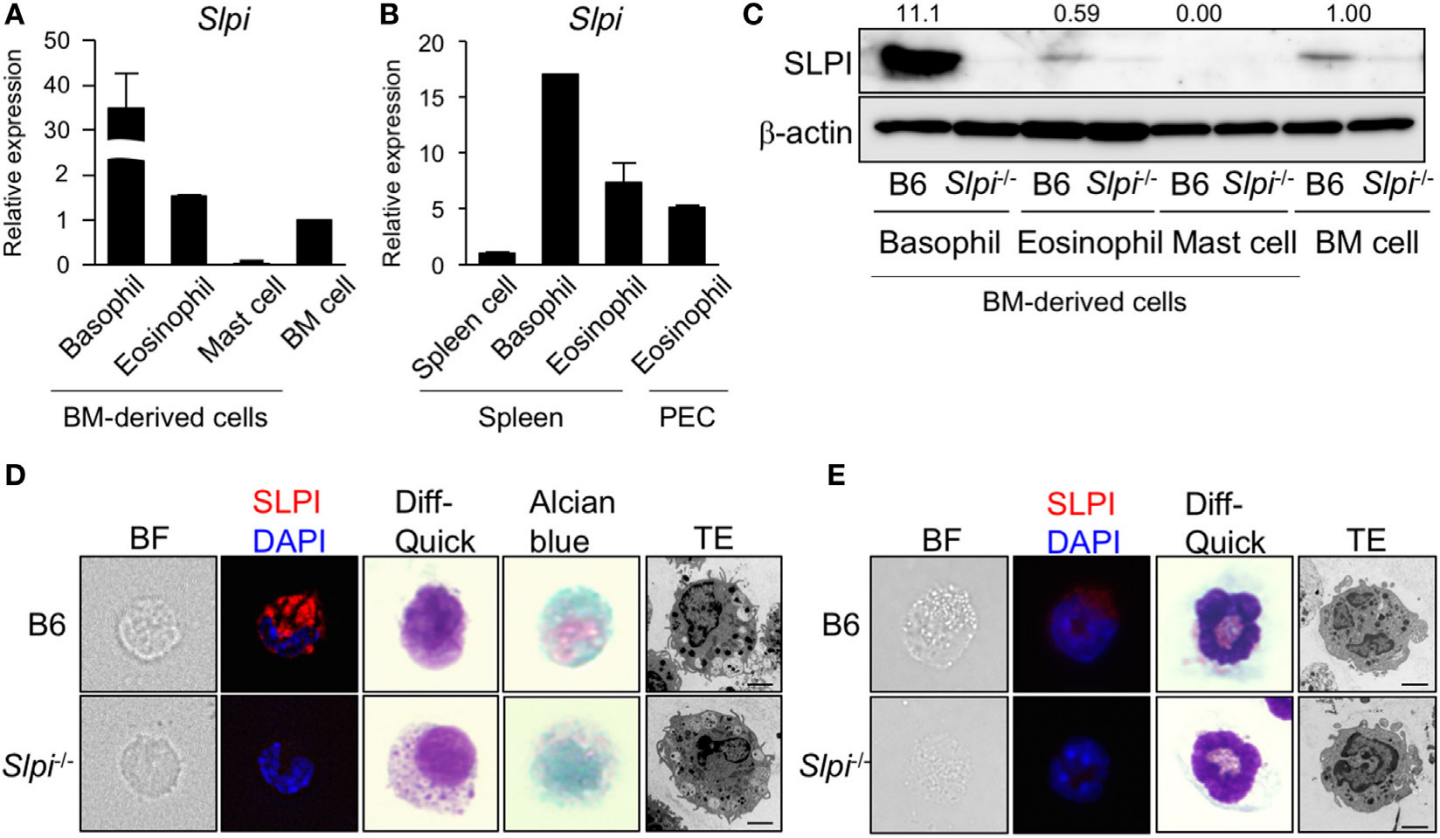
Secretory leukoprotease inhibitor (SLPI) is expressed in basophils and eosinophils but not in mast cells. **(A)** A quantitative RT-PCR of *Slpi* in s the bone marrow (BM)-derived basophils, eosinophils, mast cells, and BM cells. **(B)** Basophils and eosinophils were sorted from the spleen cells after the depletion of CD4^+^CD8^+^B220^+^ cells using magnetic separator. Eosinophils are also sorted from the peritoneal cavity (PEC). A quantitative RT-PCR of *Slpi* were shown in the indicated cells. **(C)** Immunoblotting of SLPI in the indicated cells. The data were normalized to the expression of β-actin and presented relative to the expression in BM cells. **(D,E)** Fluorescence microscopy and transmission electron microscopy (TEM) images of BM-derived basophils (BMBs) **(D)** and BM-derived eosinophils (BMEos). **(E)** from B6 and *Slpi*^−/−^ mice. Bright field (BF), SLPI (red) DAPI (blue), Diff-Quick staining, alcian blue staining, and TEM images are shown (scale bar: 2 µm). **(A,B)** Data were normalized to the housekeeping *Rps16* (mean ± SD). *n* = 4. **P < 0.01. **(C–E)** Data are representative of three independent experiments.

We first examined the expression of *Slpi* transcripts and proteins in mast cells, basophils, and eosinophils derived from BM cells. As shown in Figure 1A, murine SLPI-encoding *Slpi* transcripts were found to be abundant in BMBs. BMEos expressed a similar level of SLPI mRNA to BM cells, but SLPI mRNA was barely detected in mast cells. In addition, *Slpi* transcripts were detected in basophils and eosinophils sorted from spleen cells. SLPI mRNA was also observed in the peritoneal fluid eosinophils (Figure 1B), suggesting that SLPI is expressed in residential basophils and eosinophils.

Because these allergic effector cells are induced from BM cells by their specific growth factors for the lineage commitment, we next examined the implication of SLPI in cellular differentiation using *Slpi*^−/−^ mice (20). As shown in Figures S2A, S3A, and S4A in Supplementary Material, the proliferation curves of B6 and *Slpi*^−/−^ mice were comparable during the terminal differentiation of BM cells into mast cells, basophils, and eosinophils. There were no significant differences in the population of terminally differentiated cells between B6 and *Slpi*^−/−^ mice (Figures S2B, S3B, and S4B in Supplementary Material), suggesting that SLPI does not affect the growth of these effector cells. As shown Figure 1C, immunoblotting also demonstrated that BMBs express a large amount of SLPI protein and that the SLPI expression of eosinophils is similar to that of BM cells; however, no SLPI expression was observed in mast cells, suggesting that SLPI is expressed in basophils and eosinophils, but not in mast cells. We found that the degranulation and cytokine production in response to IgE or LPS did not differ between B6 and *Slpi*^−/−^ mast cells (Figures S2C,D in Supplementary Material). Moreover, the absence of SLPI did not affect mast cell-dependent IgE-mediated systemic anaphylaxis (Figure S2E in Supplementary Material), showing that SLPI is dispensable for the activation of mast cells.

We thus focused on the morphological analyses of BMBs and BMEos. As shown in Figure 1D, immunostaining revealed that basophils express SLPI. There were no marked differences in the Diff-Quick, or alcian blue staining patterns between B6 and *Slpi*^−/−^ BMBs. TEM demonstrated that *Slpi*^−/−^ BMBs resemble B6 BMBs morphologically, with both sharing lobulated nuclei and granules. Both B6 and *Slpi*^−/−^ BMBs showed similar expression levels of FcεRI, IL-3Rα, and ST2 (IL-33 receptor) (Figure S3C in Supplementary Material). The amount of mast cell protease (MCP)-8 and 11—granule serine proteases that are known as specific basophil markers—and the amount of lysosomal enzyme β-hexosaminidase (HEX) did not differ between B6 and *Slpi*^−/−^ BMBs (Figures S3D,E in Supplementary Material). We next explored the morphology, cell surface receptors, and granule contents in BMEos. As shown in Figure 1E, BMEos expressed SLPI at low levels, results that were consistent with the mRNA and immunoblotting data. The Diff-Quick and TEM images of B6 and *Slpi*^−/−^ BMEos were comparable. Both B6 and *Slpi*^−/−^ BMEos displayed similar expression levels of TLR4, Siglec-F, C-C chemokine receptor (CCR) 3, CD11b, and ST2 (Figure S4C in Supplementary Material). The granular enzyme, EPO, was also detected in *Slpi*^−/−^ BMEos at almost the same level as in B6 BMEos (Figure S4D in Supplementary Material). Collectively, these data showed that the basophils and eosinophils of mice express SLPI and suggested that SLPI deficiency did not affect the proliferation or the morphology of BMBs or BMEos.

### Enhanced Cytokine Production and Tryptase Activity in *Slpi*^−/−^ Basophils after IgE Stimulation

**Figure 2:**
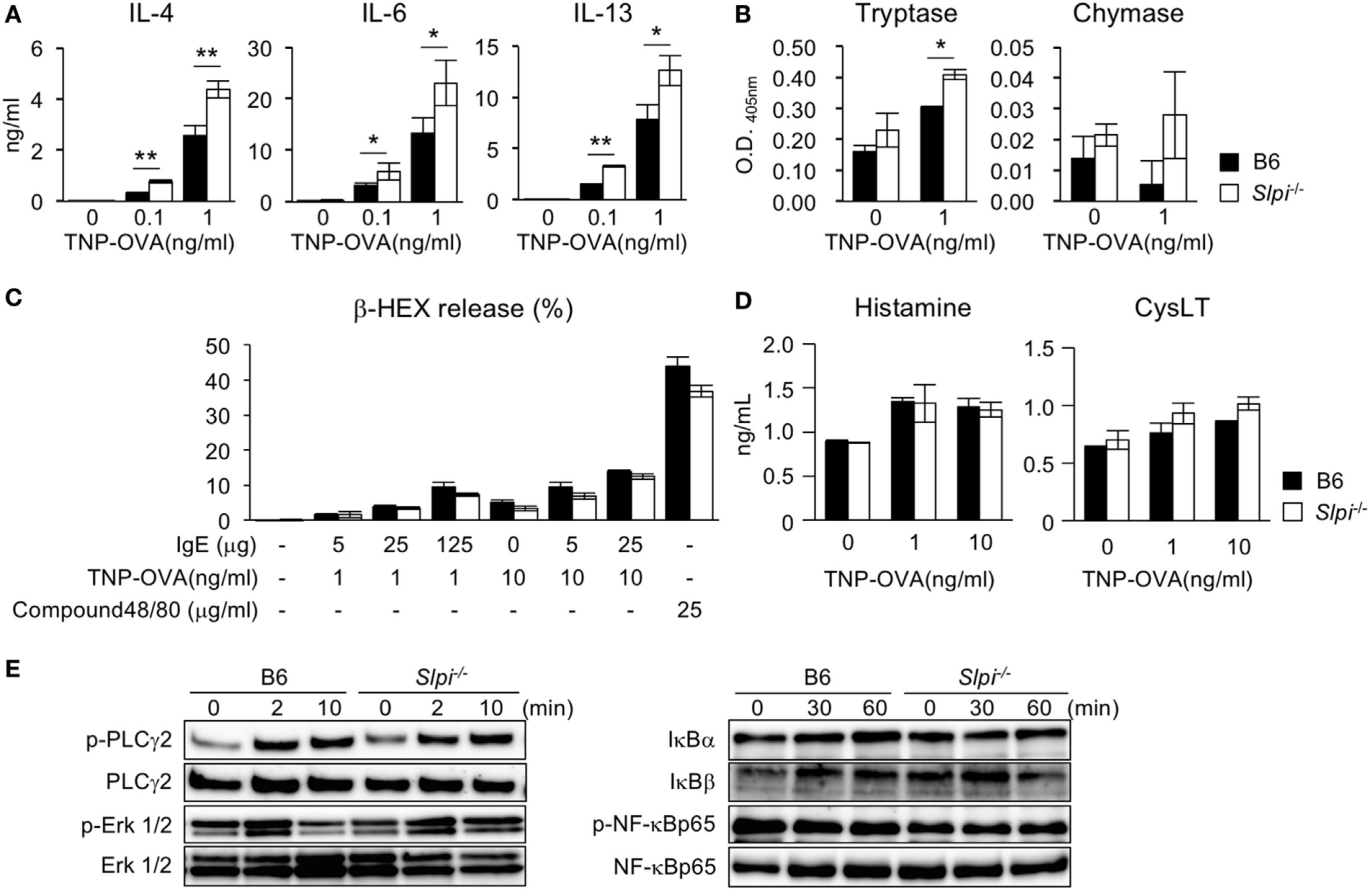
Enhanced cytokine production and tryptase activity in *Slpi*^−/−^ bone marrow-derived basophils (BMBs) after IgE stimulation. **(A–D)** B6 and *Slpi*^−/−^ BMBs were incubated with TNP-OVA for 12 h at the indicated concentrations 1 h after the administration of 5 µg/ml anti-TNP-IgE. **(A)** The interleukin (IL)-4, 6, and 13 levels in supernatants were measured by an ELISA. **(B)** The enzyme activities of tryptase (left) and chymase (right) in supernatants were determined using MeOSuc-AAPV-pNA and N-Suc-AAPF-pNA substrate, respectively. **(C)** The percentages of β-HEX released after the administration of the indicated stimulators. **(D)** The histamine and cysteinyl leukotrienes (CysLT) production in supernatants was measured by an ELISA. **(E)** B6 and *Slpi*^−/−^ BMBs were stimulated with TNP-OVA (1 ng/ml) at the indicated time, 1 h after the administration of 5 µg/ml anti-TNP-IgE. Representative immunoblots of the indicated proteins are shown. **(A–D)** Data are shown as the mean ± SEM of three different basophil cultures.

We next examined the cytokine production and serine protease activity in B6 and *Slpi*^−/−^ BMBs upon IgE stimulation. As shown in Figure 2A, *Slpi*^−/−^ BMBs produced more IL-4, 6, and 13 than B6 BMBs. *Slpi*^−/−^ BMBs also showed higher tryptase activity than B6 BMBs (Figure 2B). In contrast, there was no significant difference in the chymase activity of B6 and *Slpi*^−/−^ BMBs. As shown in Figure 2C, the release of β-HEX upon stimulation with IgE or compound 48/80 (an IgE-independent degranulator) did not differ between B6 and *Slpi*^−/−^ basophils, suggesting that SLPI does not affect basophil degranulation. Furthermore, B6 and *Slpi*^−/−^ BMBs secreted comparable levels of IgE-induced chemical mediators, histamine and cysteinyl leukotrienes (CysLT) (Figure 2D). We further investigated FcεR downstream signaling in B6 and *Slpi*^−/−^ BMBs. As shown in Figure 2E (left panel), the phosphorylation of phospholipase (PLC)-γ2 and extracellular signal-regulated kinase (Erk) 1/2 were equivalently increased in B6 and *Slpi*^−/−^ BMBs. We did not detect any elevation of NF-κB p65 phosphorylation or degradation of IκB α/β in B6 or *Slpi*^−/−^ BMBs (Figure 2E, right panel). Electrophoretic mobility shift assays (EMSAs) showed that the DNA binding activity of NF-κB was not altered upon stimulation (data not shown), suggesting that SLPI represses pathways other than NF-κB in basophils. Collectively, these results showed that SLPI inhibits IgE-mediated cytokine production and tryptase activity in basophils.

### The Depletion of Basophil SLPI Exacerbates IgE-Mediated Allergic Responses

**Figure 3:**
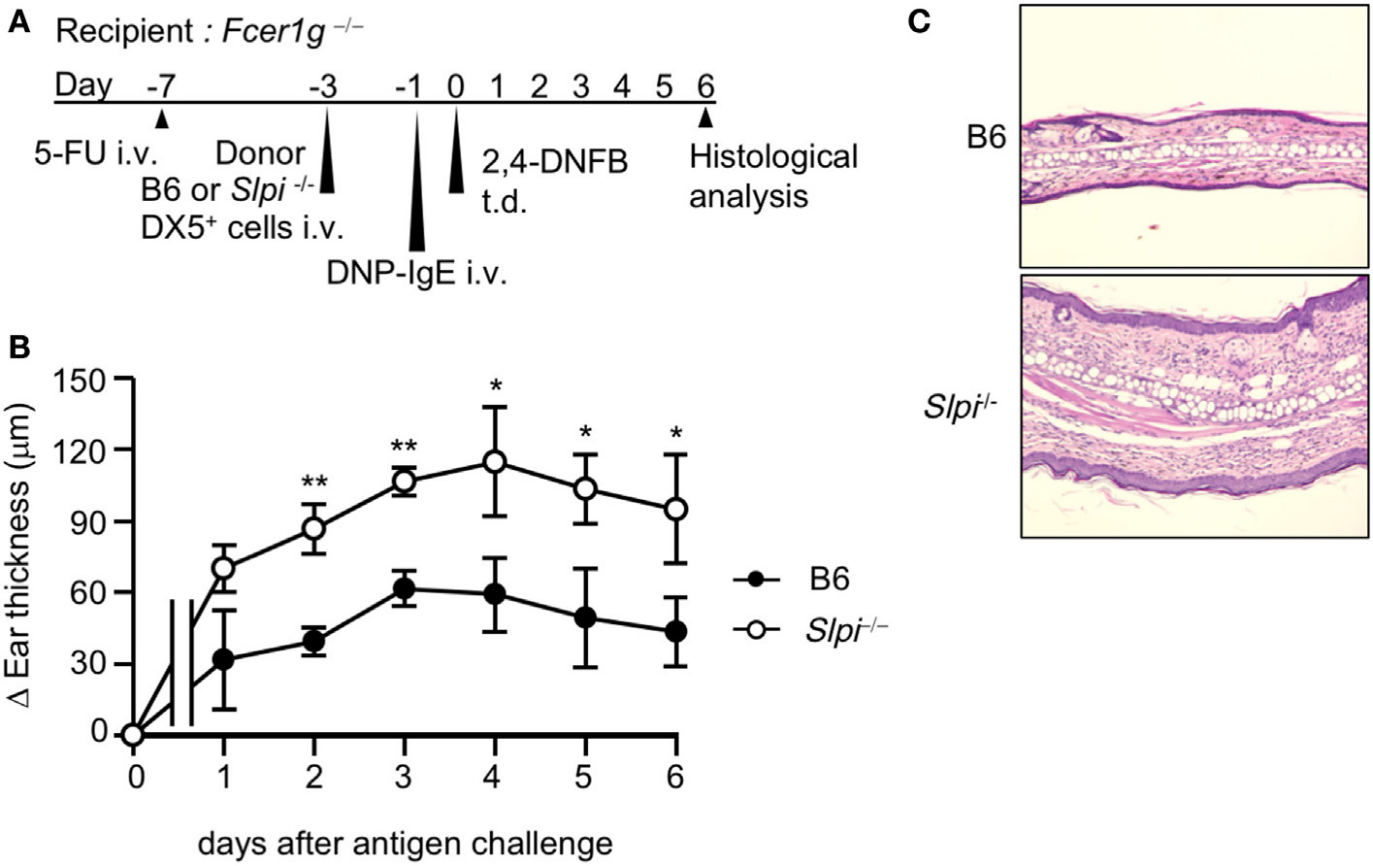
The depletion of basophil secretory leukoprotease inhibitor (SLPI) exacerbates IgE-mediated allergic responses. **(A)** The experimental protocol of IgE-mediated chronic allergic inflammation in 5-fluorouracil (5-FU)-treated *Fcer1g*^−/−^ mice adaptively transferred with B6 and *Slpi*^−/−^ DX5^+^ cells containing basophils from bone marrow (BM) cells. **(B)** The kinetics of the ear thickness after the antigen challenge are shown. **(C)** Ear specimens obtained 6 days after the antigen challenge were stained with HE. Data are representative of three separate experiments and are shown as the mean ± SD. *n* = 4–6. *P < 0.05, **P < 0.01.

The use of the DX5^+^ BM cell adaptive transfer system showed that basophils are indispensable for the development of the IgE-mediated delayed-onset cutaneous anaphylaxis reaction (IgE-CAI) in *Fcer1g*^−/−^ mice (27). Only basophils express FcεRI in the fraction of DX5^+^ BM cells (27). Thus, to investigate whether SLPI regulates basophil activation *in vivo*, we induced IgE-CAI in 5-FU-treated *Fcer1g*^−/−^ mice reconstituted with DX5^+^ BM cells, including basophils, selected from B6 or *Slpi*^−/−^ BM cells (Figure 3A). We confirmed that the population of FcεRI^+^ cells did not differ between the B6 and *Slpi*^−/−^ DX5^+^ BM cells (data not shown). As shown in Figure 3B, ear swelling in mice transferred with *Slpi*^−/−^ basophils was significantly increased in comparison to mice transferred with B6 basophils. A histopathological examination at six days after antigen challenge also demonstrated that recipients transferred with *Slpi*^−/−^ basophils showed augmented inflammatory cellular infiltration (Figure 3C). These findings demonstrated that basophil SLPI regulates the IgE-mediated allergic inflammatory responses.

### The Absence of SLPI in Eosinophils Increases IL-6 Production and Invasive Activity

**Figure 4:**
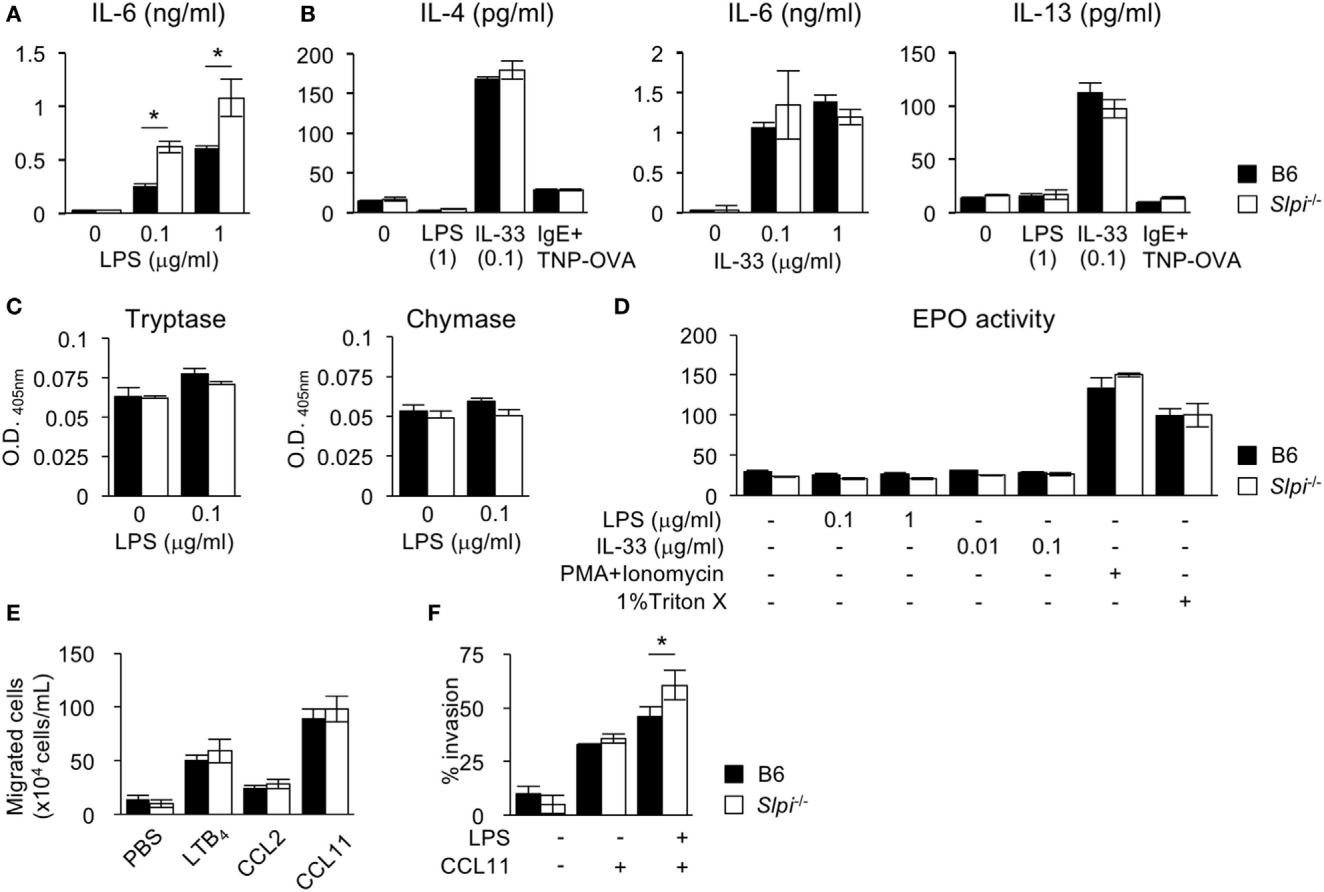
The absence of secretory leukoprotease inhibitor (SLPI) in BM-derived eosinophils (BMEos) increases interleukin (IL)-6 production and invasive activity. **(A)** I The production of IL-6 by B6 and *Slpi*^−/−^ BMEos after lipopolysaccharide (LPS) stimulation for 12 h. **(B)** B6 and *Slpi*^−/−^ BMEos were incubated with LPS (1 µg/ml) or IL-33 (0.1 µg/ml) for 12 h. BMEos were also incubated with TNP-OVA (1 ng/ml) for 12 h after the administration of 5 µg/ml anti-TNP-IgE. **(A,B)** IL-4, 6, and 13 levels in the supernatants of cells were measured by an ELISA. **(C)** The activities of tryptase and chymase prepared according to the methods described in Figure 2B. **(D)** The amounts of eosinophil peroxidase (EPO) in B6 and *Slpi*^−/−^ BMEos after the administration of the indicated stimulators. **(E)** Chemotactic assays of B6 and *Slpi*^−/−^ eosinophils by LTB_4_ (50 nM), CCL2 (50 nM), and CCL11 (10 nM). **(F)** Invasion assays using Matrigel in B6 and *Slpi*^−/−^ eosinophils upon LPS (1 µg/ml) and CCL11 (10 nM) stimulation. All of the data are shown as the mean ± SEM of three different eosinophil cultures. * *P* < 0.05.

We next investigated the cytokine production and protease activities of B6 and *Slpi*^−/−^ BMEos. IL-33, unlike LPS, is known to be a strong inducer of the Th2 cytokines IL-4 and 13 (35). We thus measured the IL-4, 6, and 13 levels after LPS or IL-33 treatment. As shown in Figure 4A, *Slpi*^−/−^ BMEos produced more IL-6 than B6 BMEos upon LPS stimulation, whereas B6 and *Slpi*^−/−^ BMEos did not secrete IL-4 or IL-13 (Figure 4B). When stimulated with IL-33, B6 and *Slpi*^−/−^ BMEos produced any or all of IL-4, 6, and 13, but there were no differences in the amounts of cytokines between B6 and *Slpi*^−/−^ BMEos (Figure 4B). In addition, the enzymatic activities of tryptase, chymase, and EPO were not increased in B6 or *Slpi*^−/−^ BMEos upon LPS or IL-33 stimulation (Figures 4C,D), showing that SLPI deficiency does not affect the serine protease activities or the degranulation responses in eosinophils, and that SLPI inhibits the IL-6 secretion induced by LPS but not that induced by IL-33.

Upon stimulation, eosinophils rapidly migrate to the inflammatory sites, and across the epithelia into tissue (1, 7–9). We therefore conducted a chemotaxis assay after stimulation with eosinophil chemotactic factors LTB4, CCL2, and CCL11. As shown in Figure 4E, the chemotactic activities in response to these chemoattractants were comparable between B6 and *Slpi*^−/−^ BMEos, indicating that SLPI does not affect the cellular migration induced by chemokines alone. We therefore performed a Matrigel invasion assay after costimulation with LPS and CCL11. As shown in Figure 4F, the invasion activity in *Slpi*^−/−^ BMEos was increased in comparison to that in B6 eosinophils. Collectively, these results suggest that SLPI regulates the LPS-mediated IL-6 production and invasion activity in eosinophils.

### SLPI Transcriptionally Regulates the MMP-9 Expression in Eosinophils

**Figure 5:**
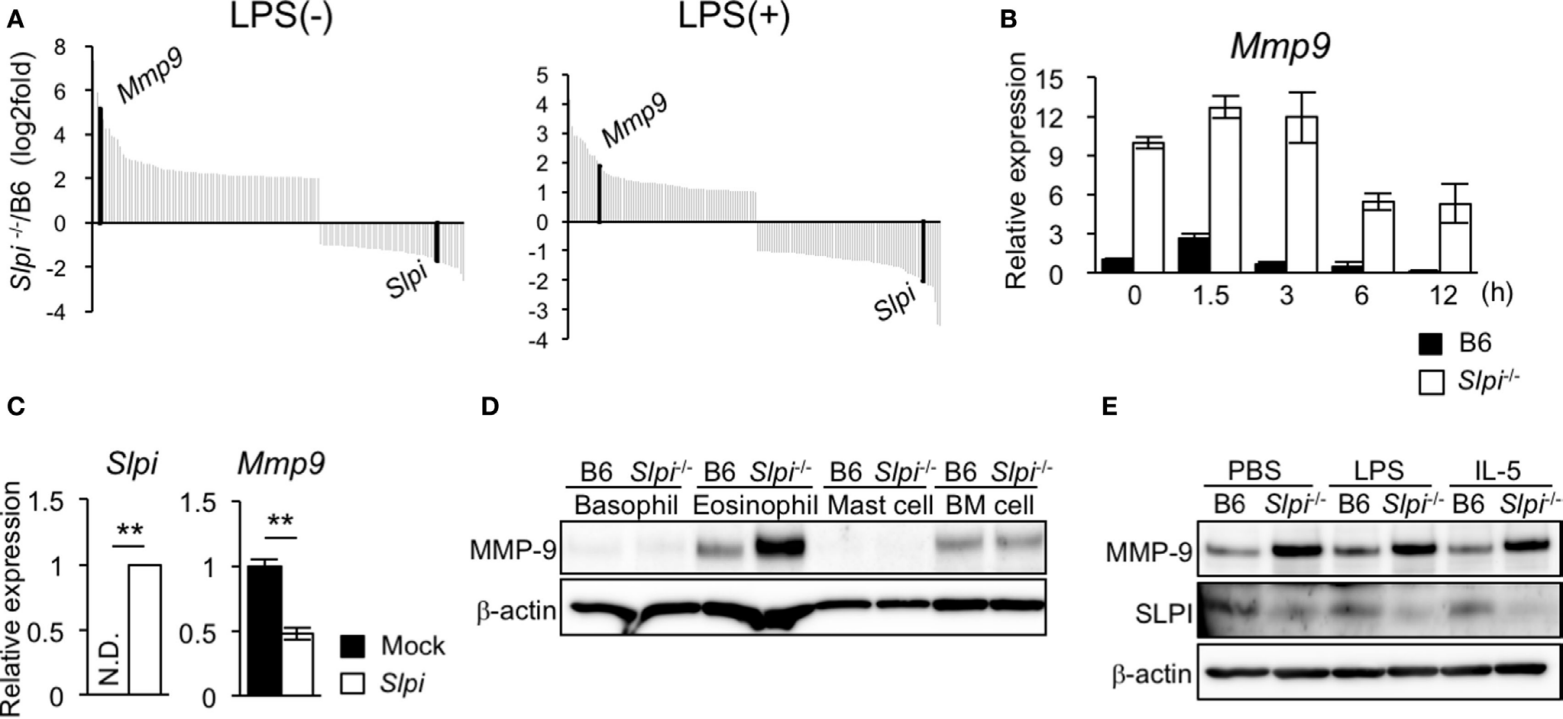
Secretory leukoprotease inhibitor (SLPI) transcriptionally regulates the metalloproteinase (MMP)-9 expression in BM-derived eosinophils (BMEos). **(A)** A DNA microarray analysis of *Slpi*^−/−^ BMEos before and 3 h after lipopolysaccharide (LPS) (1 µg/ml) stimulation. The relative expression to B6 BMEos is shown. **(B)** A qRT-PCR of *Mmp9* in B6 and *Slpi*^−/−^ BMEos after LPS (1 µg/ml) stimulation. **(C)** A qRT-PCR of *Mmp9* and *Slpi* in *Slpi*^−/−^ BMEos transfected with a plasmid carrying the *Slpi* gene. **(D)** Immunoblotting of MMP-9 in the indicated cells. **(E)** Immunoblotting of MMP-9 in B6 and *Slpi*^−/−^ BMEos after LPS (1 µg/ml) or interleukin (IL)-5 (10 ng/ml) stimulation for 6 h. **(B,C)** Data were normalized to the housekeeping *Rps16* (mean ± SD). *n* = 4. ** *P* < 0.01. **(D,E)** β-actin was used as a control. Data are representative of three separate experiments.

We investigated the gene alteration in B6 and *Slpi*^−/−^ BMEos before and after administration with LPS using DNA microarray analyses. Surprisingly, the expression of *Mmp9* in *Slpi*^−/−^ BMEos was markedly higher than that in B6 BMEos (Figure 5A). A quantitative RT-PCR confirmed that the expression of *Mmp9* transcripts in *Slpi*^−/−^BMEos was significantly higher than that in B6 BMEos before and after LPS stimulation (Figure 5B). To clarify whether the genetic disruption of SLPI affected the *Mmp9* gene in eosinophils, we introduced an *Slpi* plasmid into *Slpi*^−/−^ BMEos. As shown in Figure 5C, the *Mmp9* transcripts in the *Slpi* plasmid*-*transfected *Slpi*^−/−^ BMEos were decreased to half the level observed in the mock-transfected *Slpi*^−/−^ BMEo. Furthermore, the absence of SLPI did not alter the expression of MMP-9 protein in BMBs or BM cells (Figure 5D), suggesting that the MMP-9 expression is not impaired by the SLPI gene disruption itself, and that SLPI transcriptionally represses MMP-9 in eosinophils. On the other hand, immunoblotting showed that the expression of MMP-9 proteins was remarkably increased in *Slpi*^−/−^ BMEos at the steady state; however, the MMP-9 expression in *Slpi*^−/−^ BMEos was not enhanced after LPS treatment (Figure 5E). In addition, MMP-9 was not increased in B6 or *Slpi*^−/−^ BMEos after IL-5 stimulation (Figure 5E), inferring that IL-5 does not affect the MMP-9 expression in terminally differentiated eosinophils. It was shown that MMP-9 is critical for the migration of eosinophils though the basement membrane components (36). Since MMP-9 is increased in *Slpi*^−/−^ BMEos in the steady state, our data suggested that SLPI represses excessive eosinophil migration in part by MMP-9 expression.

### SLPI Negatively Regulates JNK1 and Elk-1 Activation, and Interacts with the JIP3 Scaffold Protein

**Figure 6:**
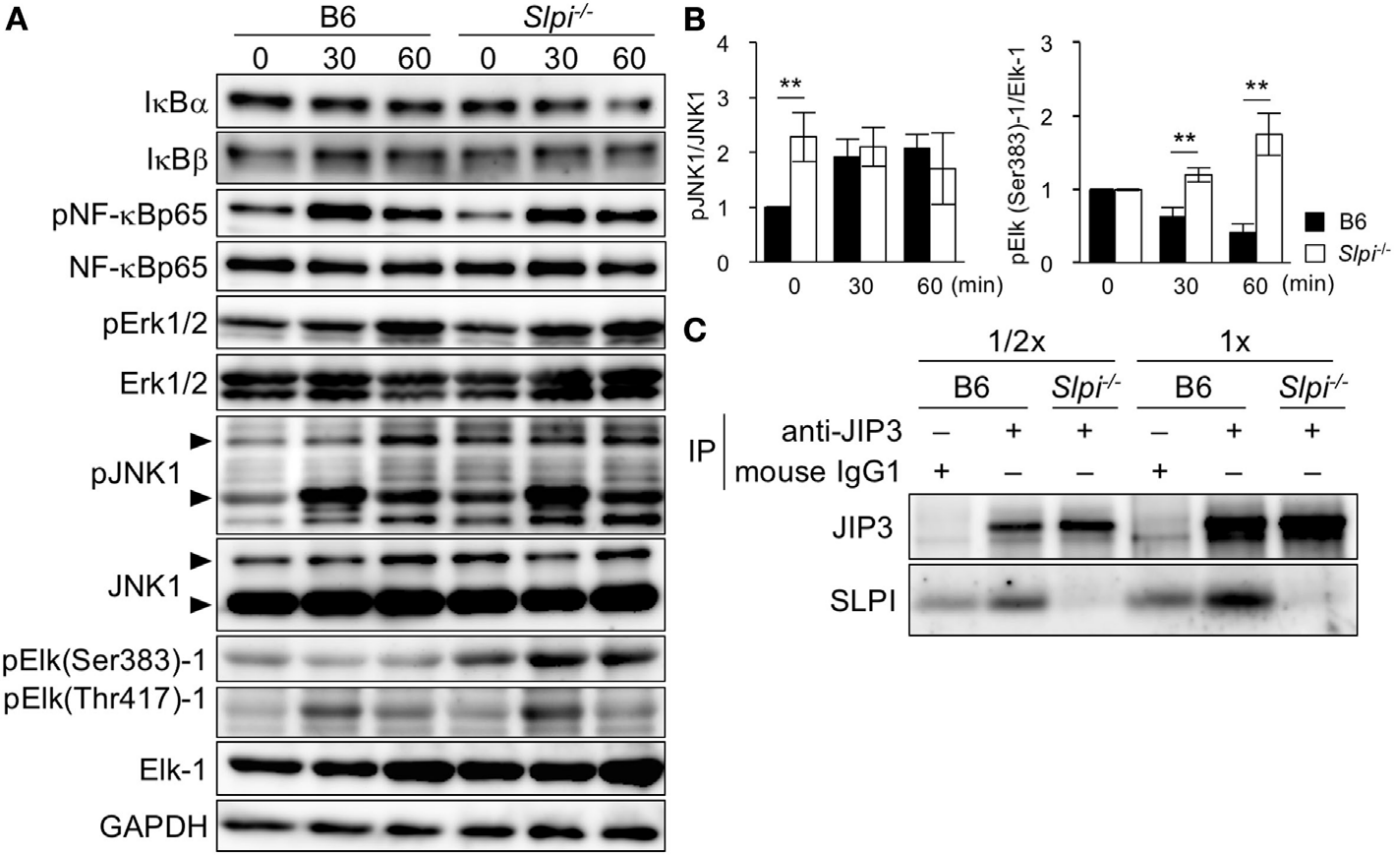
Secretory leukoprotease inhibitor (SLPI) interact with the JNK-interacting protein 3 (JIP3) scaffold protein, and negatively regulates Toll-like receptor (TLR) 4-mediated Elk-1 activation. **(A)** B6 and *Slpi*^−/−^ bone marrow-derived eosinophils (BMEos) were stimulated with lipopolysaccharide (LPS) (1 µg/ml). Immunoblots of the indicated proteins are shown. The arrows indicate the p54 and P46 isoforms of JNK1. GAPDH was used as loading and internal monitoring controls. **(B)** The relative intensities of pJNK1/JNK1 and pSer383 Elk-1/Elk-1 in B6 and *Slpi*^−/−^ BMEos were estimated by densitometric scanning with normalization to GAPDH (means ± SD). *n* = 3. *P < 0.05. **P < 0.01. **(C)** JIP3 and SLPI after the precipitation of anti-JIP3 Ab or control mouse IgG_1_ in B6 and *Slpi*^−/−^ BMEos. The loading volumes (1/2 and 1/1) are shown. **(A,C)** Data are representative of three separate experiments.

The expression of the *Mmp9* gene is induced upon the phosphorylation of several transcription factors, including NF-κB and Elk-1 (37, 38). Because SLPI is shown to regulate NF-κB activation in macrophages and neutrophils upon LPS stimulation (17, 20, 21), we investigated the TLR4-downstream signaling in eosinophils (Figure 6A). In contrast to the previous results, the degradation of IκBα/β was not clearly observed in B6 or *Slpi*^−/−^ BMEos though the degradation of IκBα was slightly but not significantly increased in *Slpi*^−/−^ BMEos in 60 min after stimulation (Figure 6A, lanes 1 and 2). The phosphorylation of NF-κB p65 in B6 and *Slpi*^−/−^ BMEos was comparable (Figure 6A, lanes 3 and 4). In addition, we were unable to detect the activation of NF-κB or CCAAT enhancer binding proteins (C/EBPs) in an EMSA (data not shown).

Lipopolysaccharide also activates MAP kinase signaling, Erk1/2 and JNK1. Both pErk1/2 and pJNK1 phosphorylate the transcriptional factor Elk-1, which has multiple serine (Ser) and threonine (Thr) phosphorylation sites (39, 40). Although pSer384 Elk-1 mainly contributes to the transcriptional activation of Elk-1 (39–41), pThr418 Elk-1 counteracts the transcriptional activation of Elk-1 itself (41). We therefore examined the phosphorylation of Erk1/2, JNK1, and Elk-1 (murine Ser383 and Thr417) upon LPS stimulation (Figure 6A). While Erk1/2 phosphorylation was comparably increased in B6 and *Slpi*^−/−^ BMEos, JNK1 was significantly phosphorylated in *Slpi*^−/−^ BMEos without stimulation (Figure 6A, lanes 7 and 8 and Figure 6B, left panel). Surprisingly, pSer383 Elk-1 was increased in *Slpi*^−/−^ BMEos, whereas Elk-1 (Ser383) was barely phosphorylated in B6 BMEos, even after LPS stimulation (Figure 6A, lanes 9 and 11 and Figure 6B, right panel). Conversely, Thr418 Elk-1 was equivalently phosphorylated in both B6 and *Slpi*^−/−^ BMEos (Figure 6A, lanes 10 and 11), inferring that SLPI represses pSer383 Elk-1 *via* association with JNK1 and/or Elk-1. We therefore performed an immunoprecipitation assay using anti-SLPI and JNK1 or Elk-1 antibodies; however, we were unable to detect any interaction between these molecules (data not shown). It was reported that the JIP family proteins function as specific scaffold proteins for JNK signaling (24, 25). Although our microarray data showed that B6 and *Slpi*^−/−^ BMEos express JIP1 and 3 genes (GSE87638), we found that JIP3, but not JIP1, is present in both eosinophils at the protein level (data not shown). JIP3 is shown to bind to the cytoplasmic domains of TLR4 with or without LPS stimulation and regulates JNK1 signaling (42). In addition, JIP3 promotes the retrograde transportation of JNK1 and lysosome (43). Because SLPI is secreted in response to various stimuli (13, 15) (Figure 5E), we examined the interaction between JIP3 and SLPI at the steady state. An immunoprecipitation assay using mouse anti-JIP3 antibody showed that JIP3 associates with SLPI (Figure 6C). These results suggested that SLPI is associated with the JIP3 scaffold protein and represses Elk-1 activation in eosinophils.

### The Disruption of SLPI Augments Eosinophil-Mediated Airway Inflammation

**Figure 7:**
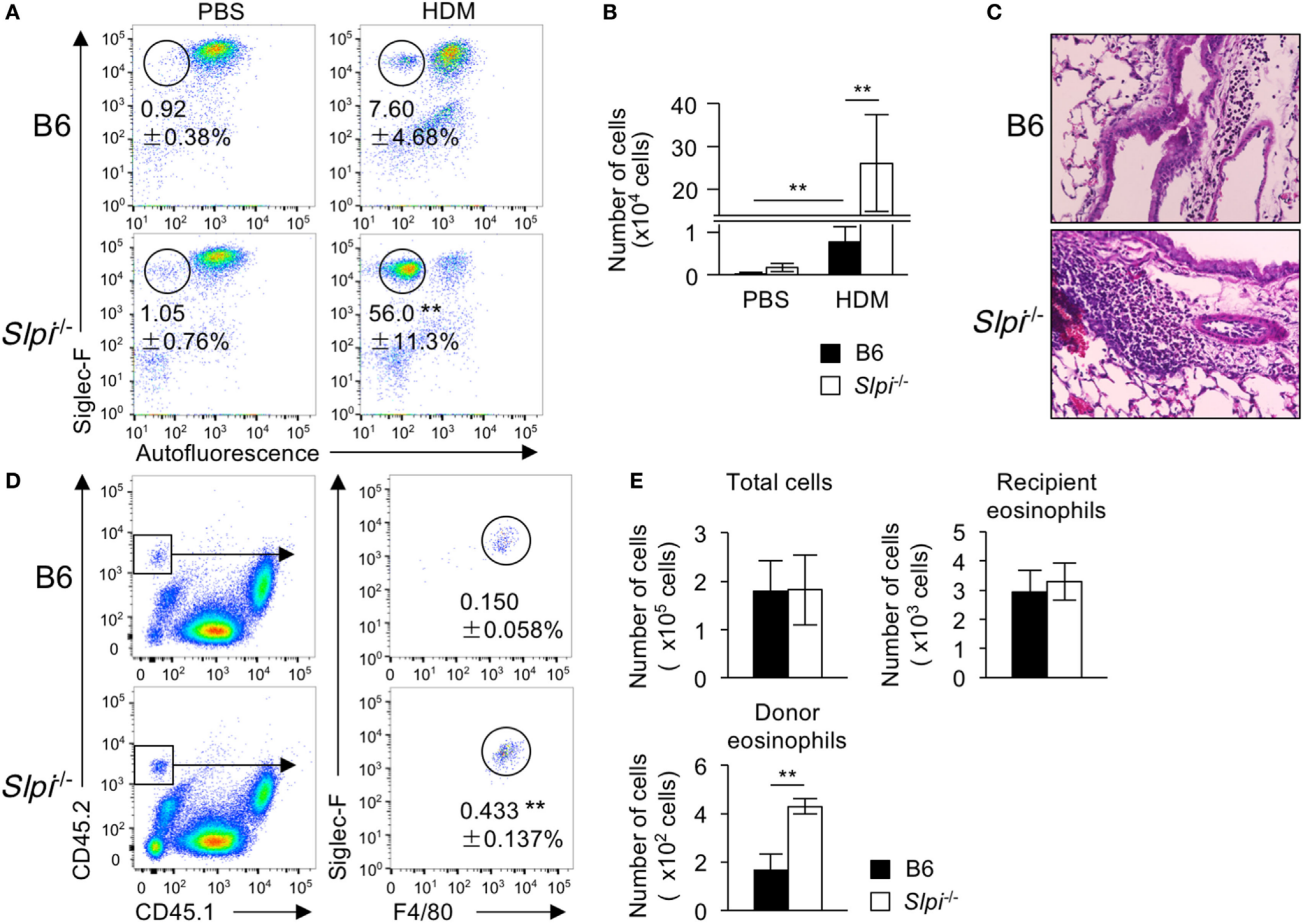
The disruption of secretory leukoprotease inhibitor (SLPI) augments eosinophil-mediated airway inflammation. **(A–C)** The house-dust mite (HDM)-induced asthmatic model. Mice were intranasally sensitized with 1 µg of HDM on Day 0 and were challenged 7 days later with exposure to 1 µg of HDM for 5 consecutive days. **(A)** The percentages of eosinophils (Siglec-F^+^ Autofluorescence^-^) among bronchoalveolar lavage fluid (BALF) cells from B6 and *Slpi*^−/−^ mice at 72 h after the last HDM challenge. **(B)** The number of eosinophils among the BALF cells on Day 14. **(C)** Lung sections from specimens obtained on Day 14 were stained with HE. **(D,E)** Chitin-induced airway inflammation using an eosinophil adaptive transfer system. CD45.2^+^ donor B6 or *Slpi*^−/−^ BMEos were intravenously transferred into allergen-challenged CD45.1^+^ recipients. **(D)** The left panel shows the population of CD45.1^+^ recipient and CD45.2^+^ donor cells among BALF cells. The right panel shows donor Siglec-F^+^ F4/80^+^ eosinophils in CD45.1 recipient mice 1 day after the antigen challenge. **(E)** The numbers of total cells, recipient eosinophils (CD45.1^+^CD45.2^−^ Siglec-F^+^ F4/80^+^cells), and donor eosinophils among BALF cells are shown. All data are representative of three separate experiments and are shown as the mean ± SD **(A,B)** *n* = 8–11, **(D,E)** *n* = 4–6. *P < 0.05, **P < 0.01.

To investigate whether or not SLPI contributes to eosinophil-mediated inflammatory responses *in vivo*, we examined HDM (house dust mite)-induced airway inflammation because TLR4 has an essential role in the HDM model (44). Mice were intranasally sensitized with small amounts of HDM (1 µg) on Day 0 and subsequently challenged on Days 7–11. After HDM, the frequency and numbers of eosinophils in the bronchoalveolar lavage fluid (BALF) cells of *Slpi*^−/−^ mice were significantly higher exposure than those in B6 mice (Figures 7A,B). A histopathological examination of a lung specimen showed that the cellular infiltration in *Slpi*^−/−^ mice was markedly greater than that in B6 mice (Figure 7C). In addition, as shown in Figure S5 in Supplementary Material, the expression of MMP-9 in the BALF were augmented in *Slpi*^−/−^ mice in comparison to B6 mice. The results were also consistent with the eosinophil numbers in the BALF.

Fungal chitin was found to induce acute eosinophilic allergic inflammation in a Myd88-dependent manner (34). We therefore used the adaptive transfer of eosinophils to examine chitin-induced airway inflammation (28). BMEos (CD45.2) were adoptively transferred in CD45.1 congenic B6 mice. Simultaneously, chitin was intranasally administered to the transferred mice. One day after the antigen challenge, we evaluated the fraction of donor (CD45.2^+^) and recipient (CD45.1^+^) Siglec-F^+^ eosinophils among BALF cells. As shown in Figures 7D,E, the numbers of the recipient eosinophils were comparable between the transferred mice, whereas the population of CD45.2^+^ donor eosinophils in mice transferred with *Slpi*^−/−^ BMEos was significantly higher than that in those that received B6 eosinophils. These results showed that SLPI regulates eosinophil-mediated airway inflammation.

## Discussion

The present study showed, for the first time, that endogenous SLPI negatively regulates the activation of basophils and eosinophils. We found that SLPI is expressed in basophils and eosinophils and that it inhibits the cytokine responses of these allergic effector cells. Notably, our data suggested that SLPI transcriptionally regulates the MMP-9 expression and suppresses Elk-1 phosphorylation *via* interaction with the JIP3 scaffold protein in eosinophils.

Our study showed that although mast cells are derived from the same common myeloid progenitors as basophils and eosinophils, they do not express SLPI. Although the regulation of the SLPI expression during differentiation remains largely unclear, a previous report showed that the expression of SLPI is upregulated in myelocytes/metamyelocytes (granulocyte progenitor cells), based on the increased expression of C/EBPα and C/EBPε (45). While C/EBPα was shown differentiation of mast cells (46). C/EBPε activates eosinophil development but is dispensable for mast cell differentiation (47). Indeed, our DNA microarray analysis provided supporting data showing that basophils and eosinophils—but not mast cells—highly express C/EBPα and C/EBPε genes (Figure S6 in Supplementary Material). Moreover, in the promoter region of the SLPI gene, the binding sites for the transcription factors associated with mast cell differentiation have not been identified (48). Thus, the transcriptional factors involved in the terminal development of mast cells may not induce the expression of SLPI; however, further studies are needed to clarify whether or not C/EBPα and C/EBPε affect the expression of SLPI.

A previous study reported that SLPI knockdown by shRNA impairs the human myeloid cell differentiation induced by granulocyte colony-stimulating factor (G-CSF) *via* the reduction of Erk and lymphoid enhancer-binding factor phosphorylation (45). In contrast, our data suggested that SLPI is dispensable for basophil and eosinophil differentiation. While G-CSF is critical for the terminal differentiation of neutrophils by association with a specific G-CSF receptor, the terminal differentiation of basophils and eosinophils is induced differently, mainly by IL-3 and IL-5, respectively, which utilize a common β-chain subunit of GM-CSF/IL-3/IL-5 receptors (49). SLPI may have a different influence on cytokine signaling transduction during cellular development.

In the present study, the cytokine production in *Slpi*^−/−^ BMBs was augmented after IgE stimulation in comparison to B6 BMBs, implying that basophil SLPI inhibits the IgE-mediated signaling cascade; however, we found no obvious evidence to show that the disruption of SLPI affects the NF-κB pathways, suggesting that SLPI controls cytokine secretion in an NF-κB-independent manner. Moreover, although MAP kinase-downstream Elk-1 has been shown to have an essential role in FcεRI-mediated mast cell activation (50), the Elk-1 phosphorylation was not elevated in either B6 or *Slpi*^−/−^ BMBs during IgE stimulation (Figure S7 in Supplementary Material), inferring that Elk-1 is scarcely involved in IgE signaling in basophils. Basophils produce various cytokines, and also express cytokine receptors for IL-3, 18, 33, and GM-CSF, which are shown to induce autocrine and/or paracrine signals in response to cellular activation *via* diverse pathways, including JAK-STAT signaling (4, 51, 52). In addition, a recent study showed that extracellular adenosine 5′-triphosphate (ATP), which is released from basophils upon IgE stimulation, induce IL-4 and 6 secretions in an autocrine manner (53). Although further studies are needed to clarify how SLPI inhibits FcεRI-mediated signals, SLPI may regulate basophil activation *via* the inhibition of signaling molecules other than the NF-κB pathway.

Our data showed that tryptase activities in *Slpi*^−/−^ BMBs were increased in comparison to B6 BMBs. It was shown that tryptase promotes acute airway hyperresponsiveness (AHR) by protease-activated receptor 2, which enhances the vascular permeability of endothelial cells (54). Mice transferred with *Slpi*^−/−^ BMBs showed an increased ear thickness; thus, basophil SLPI may regulates allergic responses in part through the suppression of tryptase activities. Conversely, like neutrophils and eosinophils, basophils are also shown to develop basophil extracellular traps (BETs) in response to IgE (55). Because *Slpi*^−/−^ mice displayed higher neutrophil extracellular trap (NET) formation (15), BET formation may be also involved in the pathological exacerbation in *Slpi*^−/−^ BMBs-transferred mice; however, further studies are needed to clarify this point. A recent study using a model of Th1-type chronic asthma showed that IFN-γ contributes to the development of AHR, and demonstrated an inverse correlation between IFN-γ and SLPI (23). While the absence of IFN-γ increased SLPI transcription, the addition of exogenous SLPI inhibited the IFN-γ secretion in AHR (23). Furthermore, *Slpi*^−/−^ mice exhibited increased cytokine production (including IFN-γ) in a model of ovalbumin-sensitized asthma (22). Although a previous study showed that IFN-γ stimulation cannot induce cytokine secretion from human basophils (56), IFN-γ may be indirectly involved with the *in vivo* phenotype of *Slpi*^−/−^ BMBs-transferred mice.

*Slpi*^−/−^ BMEos showed considerably higher MMP-9 expression (at the mRNA and protein levels) in comparison to the B6 BMEos before and after LPS stimulation. An *Slpi* plasmid transfer analysis indicated that SLPI represses MMP-9 transcription (Figure 5C), suggesting that although SLPI does not affect the eosinophil proliferation and morphology, it transcriptionally regulates the MMP-9 expression during development. Eosinophils are induced to terminally differentiate from BM cells by IL-5 after the culturing of SCF and Flt3L (7, 8, 49); however, as Figure 5E shows, we found that after IL-5 stimulation, the MMP-9 expression was not altered in either B6 or *Slpi*^−/−^BMEos, suggesting that IL-5 does not affect the MMP-9 expression in terminally differentiated eosinophils. JNK1 has been shown to promote the expression of MMP-9 though NF-κB and c-jun/c-fos complex (57). Since JNK1 phosphorylation was enhanced in steady state *Slpi*^−/−^ BMEos, it is possible that constitutive JNK1 activation may lead to the upregulation of MMP-9 in *Slpi*^−/−^ BMEos. Although further experiments are needed to clarify how SLPI affects the alteration of genes related to the expression of MMP-9, SLPI may regulate the MMP-9 expression during the eosinophil differentiation process.

Although high levels of MMP-9 proteins were observed with or without LPS treatment, *Slpi*^−/−^ BMEos showed augmented invasive activity upon stimulation, suggesting that MMP-9 augmentation is barely involved in the LPS-induced invasion of *Slpi*^−/−^ BMEos. A recent study demonstrated that the knockdown of SLPI by siRNA upregulated the expression of monocyte chemotactic protein-1 in LPS-treated periodontal ligament cells (58). Our microarray data also showed that several genes of chemokines and chemokine receptors are expressed in both B6 and *Slpi*^−/−^ BMEos (GSE87638). Moreover, it was shown that Elk-1 positively regulates the transcription of cell adhesion and migration molecules, such as connective tissue growth factor (59). Although further studies are needed, SLPI may—upon LPS stimulation—inhibit other factors (other than MMP-9) to facilitate invasion.

Elk-1 was remarkably phosphorylated at Ser384 in *Slpi*^−/−^ BMEos after LPS stimulation, suggesting that SLPI regulates the production of IL-6 *via* the suppression of Elk-1 activation. There is currently no evidence to show the direct binding of Elk-1 to the IL-6 gene promoter; however, previous studies have shown that Elk-1 plays an essential role in cytokine production *via* the induction of transcriptional factor Egr-1 (60, 61). Elk-1 has been shown to be indispensable for the expression of Egr-1 (62), and Elk-1—together with a cofactor protein, serum response factor (SRF)—immediately induces the expression of transcriptional factor Egr-1, which evokes the gene expression of cytokines including IL-6 and TNF-α (63). Although we did not obtain data showing the contribution of Elk-1 in the IL-6 production because we were unable sufficiently to reduce the Elk-1 proteins by the use of small interfering RNA in primary BMEos (data not shown), it is possible that the augmented phosphorylation of Elk-1 at Ser383 indirectly increased IL-6 production in *Slpi*^−/−^ BMEos. Conversely, pSer383 Elk-1 was barely observed in B6 BMEos, while JNK1 and Erk1/2 were phosphorylated, inferring that neither kinase promotes the Ser383 phosphorylation of Elk-1 in B6 BMEos. In addition, JNK1 phosphorylation was constitutively augmented in *Slpi*^−/−^ BMEos without stimulation, suggesting that pJNK1 is not directly connected with Elk-1 activation upon stimulation. On the other hand, JIP3 also has been shown to act as an essential transporter of pJNK1 *via* interaction with motor complexes (43), implying that the SLPI–JIP3 interaction regulates pJNK1 transportation; however, we were clearly unable to detect pJNK1 proteins associated with JIP3 immunoprecipitates under our experiment conditions (data not shown). It was shown that JIP proteins form complexes with multiple proteins, including JNK signaling cascade (64). A recent report demonstrated that Elk-1 phosphorylation at Ser384 occurs faster than that at Thr418 after stimulation (41). Although further studies are needed to clarify the detailed mechanism how SLPI regulates the interaction between JIP3 and pJNK1, SLPI may indirectly interfere with the rapid access of pJNK1 to the Elk-1 phosphorylation site of Ser384 *via* an association with JIP3.

In summary, we highlighted that SLPI is an endogenous negative regulator in basophils and eosinophils. Our data also suggested a new role of SLPI in the regulation of TLR4 signaling, which could regulate excessive Elk-1 activation in eosinophils. Basophils are essential for allergic cutaneous and airway inflammation (4, 5, 65). In addition to allergies, excessive eosinophil activation leads to chronic inflammatory diseases (7, 8, 49). The regulation of the SLPI pathway may therefore counteract basophil-and/or eosinophil-associated disorders.

## Ethics Statement

All of the studies were approved by the Animal Studies Committee at Kanazawa Medical University and Tohoku Medical and Pharmaceutical University.

## Author Contributions

SM and AN designed the experiments. SM, TY-W, KT, TS, MS, and AN performed the experiments and analyzed the data. TT, TK, and TN provided the materials. SM, TS, MS, TT, TK, TN, and AN wrote and reviewed the manuscript.

## Conflict of Interest Statement

The authors declare that the research was conducted in the absence of any commercial or financial relationships that could be construed as a potential conflict of interest.

## Footnotes

^1^http://www.bioconductor.org/.

^2^http://www.r-project.org/.

^3^http://www.ncbi.nlm.nih.gov/geo/.
