# Supplementary material for: Identification of Secretory Leukoprotease Inhibitor As an Endogenous Negative Regulator in Allergic Effector Cells
Source: Front Immunol. 2017 Nov 13;8:1538. doi: 10.3389/fimmu.2017.01538 (PMC5693852; doi:10.3389/fimmu.2017.01538)
Supplement: Supplementary file 1 [file data_sheet_1.docx]

***Supplementary Material***

**Identification of Secretory Leukoprotease Inhibitor as an endogenous negative regulator in allergic effector cells**

Shintaro Matsuba, Toshiki Yabe-Wada, Kazuya Takeda, Tetsuya Sato, Mikita Suyama, Toshiyuki Takai, Toshiaki Kikuchi, Toshihiro Nukiwa, Akira Nakamura*****

*** Correspondence:** Akira Nakamura: aki-n@tohoku-mpu.ac.jp

**Supplemental Figures**

**Supplemental Figure 1 (A)** Splenic FcεRI^+^ Basophils and Siglec-F^+^ eosinophils were sorted from spleen cells after the depletion of CD4^+^, CD8^+^, and B200^+^ cells. Upper-left panel shows the expression of Siglec-F^+^FcεRI^+^ cells in CD4-CD8-B200-depleted spleen cells. FcεRI^+^ cells and Siglec-F^+^ cells are isolated from CD4-CD8-B200-depleted spleen cells by cell sorter. Upper-right panel shows FceRI^+^DX5^+^ fractions in sorted cells (basophils; purify≥90%). Lower-right panels shows Siglec-F^+^ fractions in sorted cells (eosinophils; purify≥90%). **(B)** Siglec-F^+^ eosinophils were sorted from the fluids of the peritoneal cavity (PEC). Left panel shows the subset of Siglec-F^+^FcεRI^+^ cells in PEC. Right panel shows sorted Siglec-F^+^ cells (eosinophils; purify≥90%).

**Supplemental Figure 2.** SLPI is dispensable mast cell differentiation and activation. **(A)** Proliferative kinetics of BM cells isolated from B6 and *Slpi*^-/-^ mice during mast cell differentiation. BM cells were cultured with IL-3 for 35 days. Numbers indicate the mean ± SEM (*n*=3 per group). **(B)** The fraction of c-kit^+^ FcεRI^+^ cells (mast cells) from B6 and *Slpi*^-/-^ BM cells on Day 35. Data are representative of three independent experiments. **(C, D)** BM-derived B6 and *Slpi*^-/-^ mast cells were incubated with TNP-OVA for 12 h at the indicated concentrations 1 h after administration of 5 µg/ml anti-TNP-IgE, or were stimulated with LPS at the indicated concentrations. Percentages of β-HEX release **(C)**, and levels of IL-6 **(D)** from B6 and *Slpi*^-/-^ mast cells are shown. Data show the mean ± SEM of three different cell cultures. **(E)** Changes in rectal temperature of mice during IgE-induced systemic anaphylaxis. B6 (filled circle) and *Slpi*^-/-^ (open circle) mice intravenously received 1.0 mg TNP-OVA 24 h after administration with anti-TNP IgE. The monitoring of rectal temperature was started at the time of antigen injection using a rectal probe coupled to a digital thermometer (Natsume Seisakusyo). Data are expressed as mean ± SD (*n* = 3).

**Supplemental Figure 3.** Differentiation of basophils derived from BM cells of B6 and *Slpi*^-/-^ mice. **(A)** Proliferative kinetics of BM cells isolated from B6 and *Slpi*^-/-^ mice during basophil differentiation. BM cells were cultured with IL-3 for 12 days. **(B)** The fraction of c-kit^+^ FcεRI^+^ cells from B6 and *Slpi*^-/-^ BM cells on Day 12 (left panel). Subsets of DX5^+^ FcεRI^+^ cells (basophils) after magnetic separation by anti-DX5 mAb (right panel). **(C)** Representative histograms of the indicated surface antigens from B6 (solid line) or *Slpi*^-/-^ (dotted line) BM-derived basophils (BMBs). The shaded area shows the isotype control. **(D)** Immunoblotting of MCP8, MCP11, and SLPI in whole-cell lysates of B6 and *Slpi*^-/-^ BMBs. β-actin was used as loading and internal monitoring controls. **(E)** The amounts of β-hexosaminidase from B6 and *Slpi*^-/-^ BMBs. **(A)** The numbers indicate the mean ± SEM (n=3 per group). **(B–D)** Data are representative of three independent experiments. **(E)** Data show the mean ± SEM of three different cell cultures obtained from different mice.

**Supplemental Figure 4.** Differentiation of eosinophils derived from BM cells of B6 and *Slpi*^-/-^ mice. **(A)** Proliferative kinetics of BM cells isolated from B6 and *Slpi*^-/-^ mice during eosinophil differentiation. BM cells were cultured in the presence of SCF and Flt3L for 4 days. Thereafter, the medium was replaced with IL-5-containg medium. (up to Day 14) **(B)** The fraction of Siglec-F^+^ cells (eosinophils) from B6 and *Slpi*^-/-^ BM cells on Day 12. **(C)** Representative histograms of the indicated surface antigens from B6 (solid line) or *Slpi*^-/-^ (dotted line) BM-derived eosinophils (BMEos). The shaded area shows the isotype control. **(D)** The amounts of eosinophil peroxidase (EPO) in B6 and *Slpi*^-/-^ BMEos. **(A)** The numbers indicate the mean ± SEM (n=3 per group). **(B, C)** Data are representative of three independent experiments. **(D)** Data show the mean ± SEM of three different cell cultures obtained from different mice.

**Supplemental Figure 5.** SLPI deficiency increased the MMP-9 expression in an HDM-induced asthmatic model. Immunoblots of MMP-9 in the BALF from B6 and *Slpi*^-/-^ mice on day 14. Left panel shows the relative intensities of MMP-9 in B6 and *Slpi^-^*^/-^ BMEos. The relative intensities were quantified with respect to the mean intensity of B6 MMP-9 set to 1.0. Right panel shows the number of eosinophils from the same samples. Data are expressed as mean ± SD (*n* = 5). * P < 0.05.

**Supplemental Figure 6.** Gene expression of the C/EBP family with DNA microarrays in B6 BMBs and BMEos. The gene expression of the C/EBP family with DNA microarrays in B6 basophils and eosinophils. The relative expression against BM-derived B6 mast cells is shown. The scale represents the intensity of gene expression (log2 fold).

**Supplemental Figure 7.** Elk-1 phosphorylation in B6 and B6 and *Slpi*^-/-^ BMBs after IgE stimulation. B6 and *Slpi*^-/-^ BMBs were stimulated with TNP-OVA (1 ng/ml) at the indicated time 1 hour after administration of 5 µg/ml anti-TNP-IgE. Representative immunoblotting of pElk-1 (Ser383) and Elk-1 are shown.
